# Concordance Analysis of Lower Third Molar Surgery Classifications: A Comparative Study

**DOI:** 10.3390/dj12060167

**Published:** 2024-06-03

**Authors:** Selene Barone, Francesco Bennardo, Marianna Salviati, Vincenzo Cosentino, Riccardo Finamore, Vincenzo Greco, Antonio Madonna, Anna Procopio, Alessandro Antonelli, Amerigo Giudice

**Affiliations:** 1 Department of Health Sciences, School of Dentistry, Magna Graecia University of Catanzaro, 88100 Catanzaro, Italy; selene.barone@unicz.it (S.B.); francesco.bennardo@unicz.it (F.B.); marianna.salviati@studenti.unicz.it (M.S.); vincenzo.cosentino@studenti.unicz.it (V.C.); riccardo.finamore@studenti.unicz.it (R.F.); vincenzo.greco001@studenti.unicz.it (V.G.); antonio.madonna@studenti.unicz.it (A.M.); a.giudice@unicz.it (A.G.); 2 Department of Experimental and Clinical Medicine, Magna Graecia University of Catanzaro, 88100 Catanzaro, Italy; anna.procopio@unicz.it

**Keywords:** lower third molar, difficulty assessment, lower third molar classifications, tooth impacted, inferior alveolar nerve, risk assessment, tooth extraction

## Abstract

The high frequency and complexity of mandibular third molar (M3M) surgery have led several authors to the development of classification systems for better evaluation and management in oral surgery. This study compared the classifications of Juodzabalys and Daugela et al. (JD), Sammartino et al., Chang et al., Jhamb et al., Maglione et al., and Nemsi et al. to understand the concordance between the scores of M3M surgery. Two types of analysis were conducted: the relationship between the M3M and the inferior alveolar nerve (IAN), and the overall difficulty score based on the tooth’s angulation and its spatial position with the adjacent structure. The analysis of the classifications on the relationship between M3M and IAN resulted in a concordance of 26.1%. In the pairwise comparisons, the classifications of Nemsi et al. and Jhamb et al. showed the highest concordance of 59.5%. Analyzing the total scores difficulty, the JD et al., Chang et al., and Sammartino et al. classifications demonstrated a concordance level of 25.5%. A pairwise assessment revealed a higher concordance degree between the classifications of Sammartino et al. and Chang et al. (57.4%). The results highlight the limits in establishing a comprehensive and objective classification for the surgical difficulty of M3M, possibly attributed to variations in the methodology for computing total scores. An objective, automated, and non-operator-dependent classification method for assessing the surgical difficulty of M3M is still needed.

## 1. Introduction

The surgical extraction of the lower third molar is one of the most common procedures performed in oral and maxillofacial surgery. This type of surgery carries the possibility of several complications, including swelling, bleeding, pain, trismus, infection, and fracture [1,2,3,4,5]. One of the most feared complications is IAN injury during the surgical procedures: the known risk of a transient IAN injury after M3M surgery ranges from 0.6 to 5.3%, while the risk of permanent IAN damage is less than 1% [6,7]. In the planning of M3M surgery, it is important to minimize the potential risks and maximize the preoperative analyses by evaluating clinical and radiological records to define the anatomical characteristics of third molars and their relationships with the adjacent anatomical structures.

The definition of a detailed treatment plan allowed the reduction of postoperative sequelae during M3M surgery. For this reason, various classifications have been proposed by several authors [8,9,10,11,12,13]. The most used classifications have been carried out based on bidimensional (2D) or three-dimensional (3D) radiological investigations. Among the classifications based on 2D investigations, those of Winter, and Pell and Gregory are the most commonly used [14]. In any case, although the Orthopantomogram (OPG) represents the most widely used radiological investigation in the planning of M3M surgery, this imaging technique has limitations related to the 2D nature of the image, which does not allow for a precise assessment of the relationship between the M3M and the adjacent anatomical structures. Moreover, these images may exhibit overlaps or be distorted, leading the operator to a misinterpretation of the surgical intervention planning [15,16]. In order to overcome the limitations of OPG, the introduction of 3D imaging has increased in recent years. Cone-beam computed tomography (CBCT) offers several advantages over OPG as it delivers a lower radiation dose with a high spatial resolution. Previous studies have demonstrated that CBCT has proven to be more accurate in determining the relationship between the M3M and the IAN enhancing pre-operative surgical risk assessment [17], as well as ensuring a better qualitative and quantitative assessment of the bucco-lingual and mesio-distal relationships of the wisdom tooth with the adjacent structures. In the field of lower third molar surgery, several classifications have been developed using CBCT scans to evaluate and categorize the difficulty of procedures. Each study has adopted a unique approach, considering a set of specific parameters that have not been uniformly included in all the existing classifications. Nevertheless, it is important to note that, none of the proposed classifications have received formal validation through comprehensive clinical studies or consolidated scientific consensus. Consequently, to date, there remains a lack of a universally accepted model for assessing the difficulty of surgical procedures related to lower third molars. 

This study aimed to compare the 3D classifications proposed by Juodzabalys and Daugela et al. (JD) (2013) [13], Sammartino et al. (2017) [11], Chang et al. (2020) [8], Jhamb et al. (2009) [10], Maglione et al. (2015) [9], and Nemsi et al. (2017) [12], with the purpose to understand their concordance degree on the definition of the lower third molar surgery difficulty score.

## 2. Materials and Methods

### 2.1. Study Design

This study was designed as a retrospective single-center cross-sectional investigation. It was conducted in accordance with the Declaration of Helsinki and approved by the Regional Ethics Committee (n. 122/2023). 

### 2.2. Study Sample

The digital archive of the Oral Surgery service was reviewed, and all the Computed Tomography (CT) or CBCT scans were screened from January to September 2023. The study sample consisted of 3D images with the following inclusion criteria: (1) complete apexification of the tooth (stage H by Donald B. Shumaker, D.D.S., M.S.) [18]; (2) the presence of one or both the lower third molars; (3) radiographic investigations with a good resolution and a good visibility of the circumferential area of the lower third molars. On the other hand, the exclusion criteria involved: (1) the absence of the lower second molar; (2) local radiotransparent or radiopaque lesions in the surrounding areas of the lower third molar; (3) a history of mandibular trauma; (4) systemic pathology affecting the bone tissue; (5) incomplete or poor image quality radiographic examinations; (6) non-complete apexification of the roots.

### 2.3. Data Collection Method 

After data anonymization, the analysis of the Digital Imaging and Communications in Medicine (DICOM) files allowed the identification and classification of the M3M according to six different classifications: Maglione et al. [9], Nemsi et al. [12], Jhamb et al. [10], JD et al. [13], Sammartino et al. [11], and Chang et al. [8] (Table 1, Table 2, Table 3, Table 4, Table 5 and Table 6).

Four investigators (VC, VG, AM, and RF) separately conducted the lower third molar analysis, and the evaluation was repeated one month after the first one for 15% of the sample. Any disagreements between the four authors were discussed and judged by an expert author (AG). 

### 2.4. Study Outcomes

**Figure 1:**
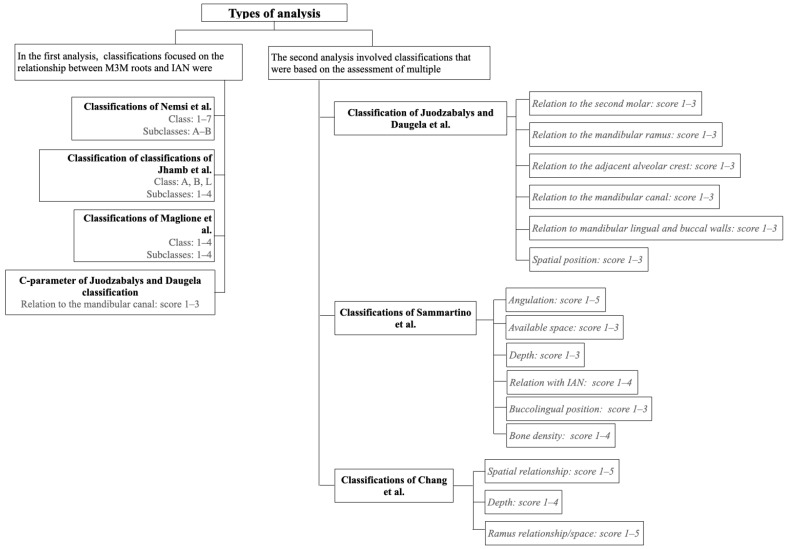
Summary diagram of the analyses conducted and the classifications included with the respective parameters [8,9,10,11,12,13].

To perform an accurate comparison between the classifications included in the study, two types of analysis were conducted (Figure 1). 

In the first analysis, only the classifications focused on the relationship between the M3M roots and the IAN were considered. The classifications of Maglione et al. [9], Nemsi et al. [12], Jhamb et al. [10], and the C-parameter of JD et al. [13] were compared. To compare the differently structured classifications, a process of equalization of scores was carried out: classes/subclasses with minimal differences were combined to obtain a total of four levels in each classification (Table 7).

In the second analysis, the involved classifications were based on the assessment of multiple parameters (JD et al. [13], Sammartino et al. [11], and Chang et al. [8]). Sammartino et al. [11] defined a final surgical difficulty score based on three levels, while JD et al. [13] and Chang et al. [8] distinguished four levels of difficulty: an equalization process of the scores was carried out as shown in Table 8. 

### 2.5. Statistical Analysis 

The data were collected and reported in a single Excel database (version 16.85, Microsoft, Redmond, WA, USA). The statistical analyses were performed using R Studio software (version 4.3.0, 250 Northern Ave, Suite 420, Boston, MA, USA, 02210). The descriptive statistics reported the mean and standard deviation for the continuous quantitative variables, as well as the absolute frequencies and percentages for the categorical variables. To assess the degree of agreement between the total scores of the analyzed classifications, the Chi-squared test was performed, setting α = 0.05 as the significance level. 

## 3. Results

**Figure 2:**
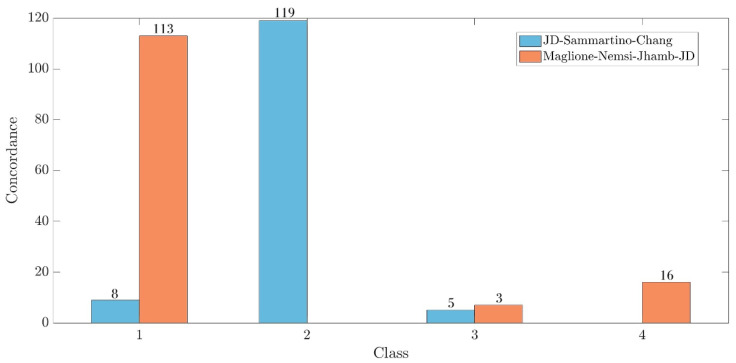
Histogram of the concordance degree among the classifications that evaluated the relationship between the M3M roots and the IAN (JD et al. [13], Sammartino et al. [11], and Chang et al. [8]), and those based on the assessment of multiple parameters (JD et al. [13], Sammartino et al. [11], and Chang et al. [8]).

The study sample included 521 radiographic images that featured a M3M. The intra- and inter- observer agreement were >88%. The descriptive analysis is reported in Table 9, for the surgical difficulty score and for the evaluation of the relationship between the M3M and the IAN. Regarding the difficulty score concerning the relationship between the M3M and the IAN, the analysis of the classifications by Maglione et al. [9], Nemsi et al. [12], JD et al. [13], and Jhamb et al. [10] resulted in concordance for 136 cases (26.1%). Particularly, there was an agreement for 113 images of grade 1, none of grade 2, three of grade 3, and 16 of grade 4 (Figure 2).

**Figure 3:**
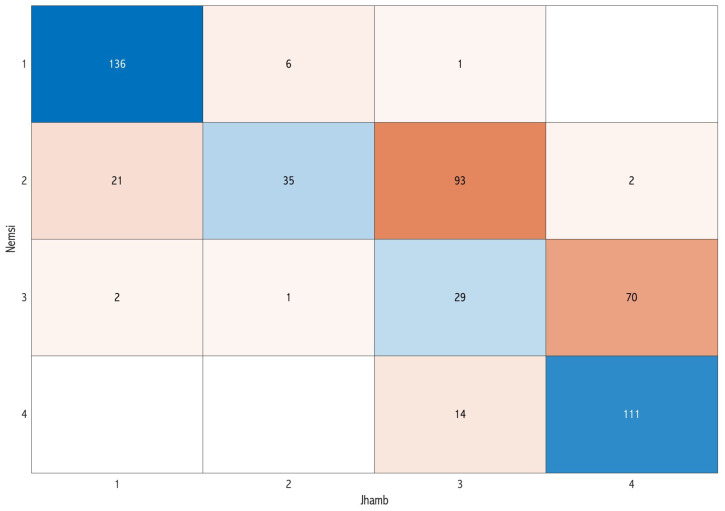
Confusion matrix comparing the classification of Nemsi et al. [12] and Jhamb et al. [10] in the pairwise assessment: the diagonal represents the concordance cases of the difficulty score.

**Figure 4:**
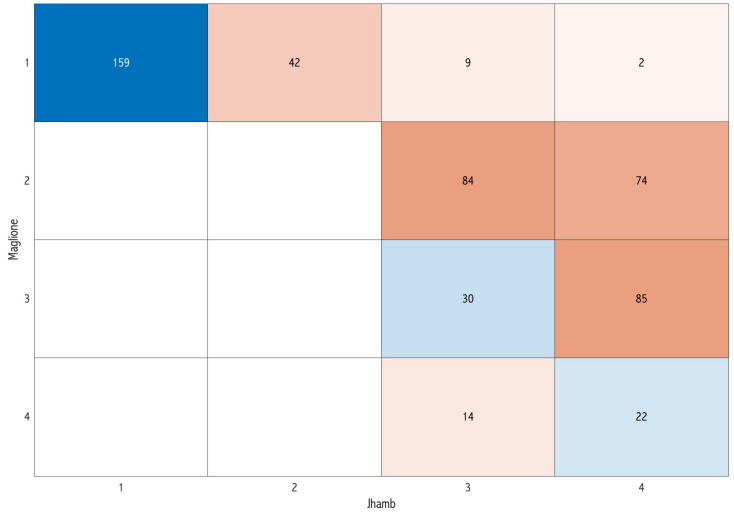
Confusion matrix comparing the classification of Maglione et al. [9] and Jhamb et al. [10] in the pairwise assessment: the diagonal represents the concordance cases of the difficulty score.

In the pairwise comparisons, the classifications of Nemsi et al. [12] and Jhamb et al. [10] showed the highest concordance at 59.5% (Figure 3), while a lower level of concordance was observed in the comparison between Maglione et al. [9] and Jhamb et al. [10] (40%) (Figure 4).

**Figure 5:**
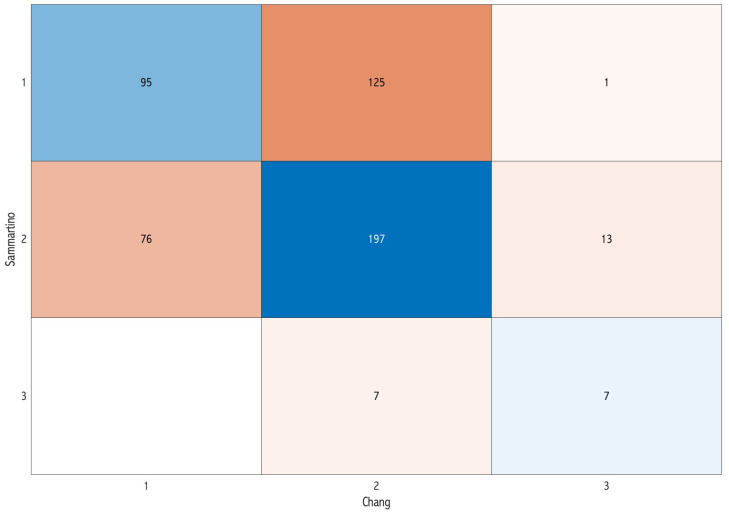
Confusion matrix comparing the classification of Sammartino et al. [11] and Chang et al. [8] in the pairwise assessment: the diagonal represents the concordance cases of the difficulty score.

**Figure 6:**
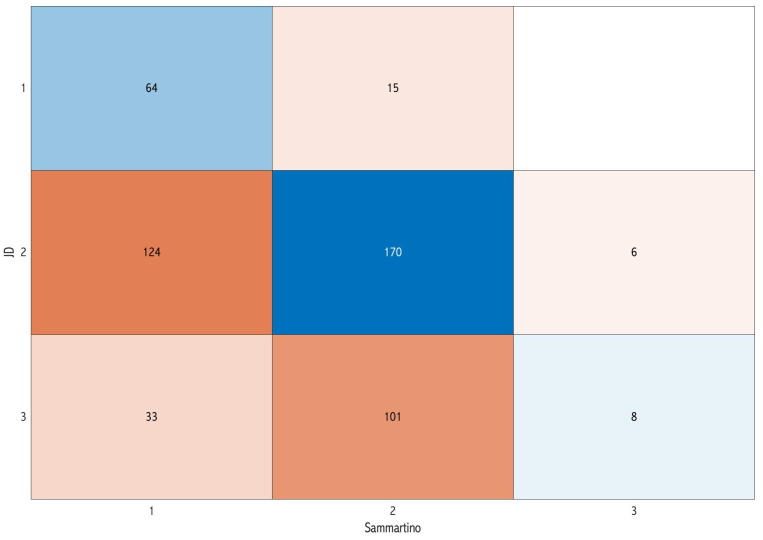
Confusion matrix comparing the classification of Sammartino et al. [11] and JD et al. [13] in the pairwise assessment: the diagonal represents the concordance cases of the difficulty score.

**Figure 7:**
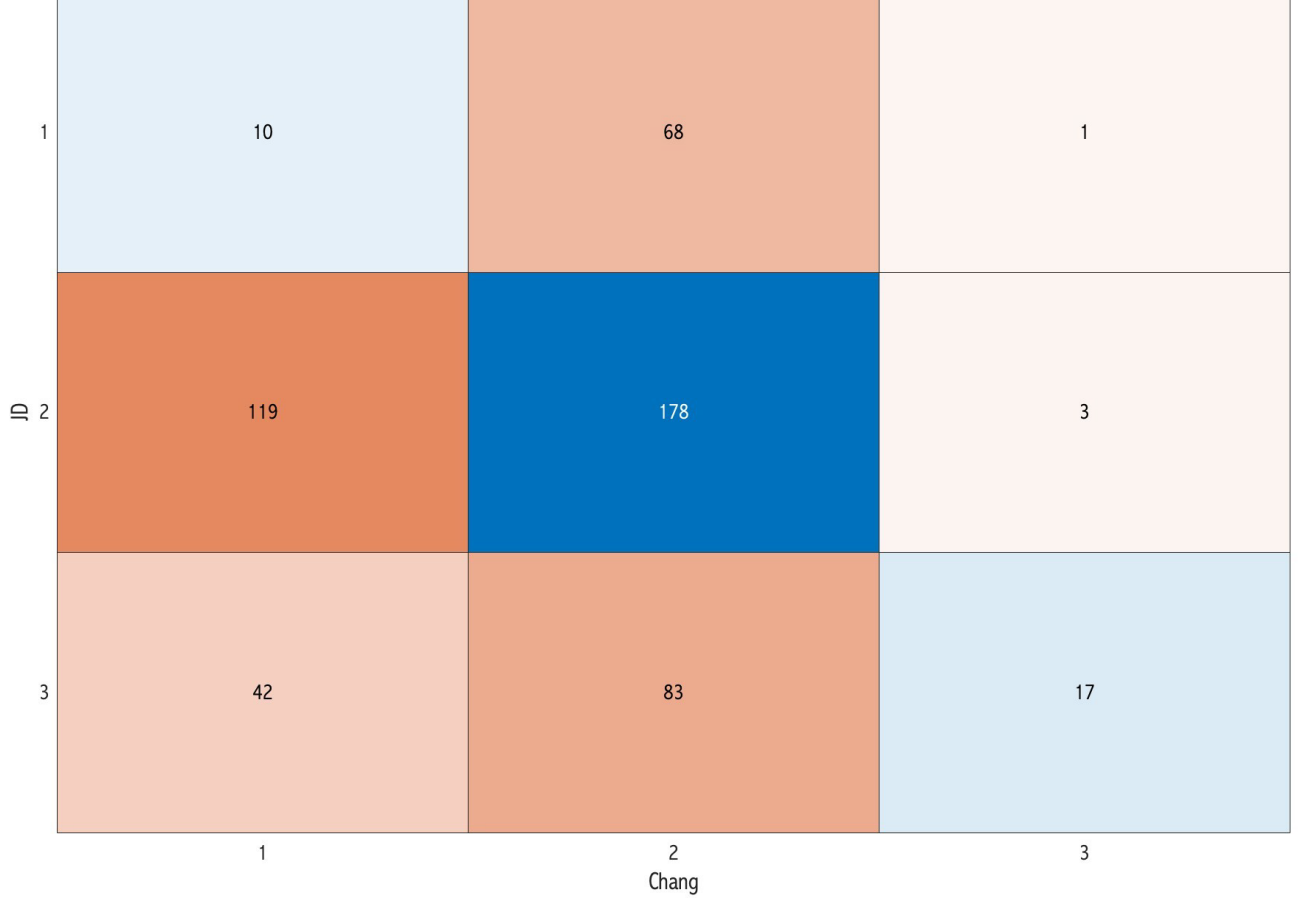
Confusion matrix comparing the classification of Chang et al. [8] and JD et al. [13] in the pairwise assessment: the diagonal represents the concordance cases of the difficulty score.

Concerning the overall difficulty score, a total of 133 images (25.5%) exhibited concordance among the three classifications. Specifically, eight images were unanimously classified as having a simple score of surgical difficulty, 119 as moderate, and five as difficult (Figure 2). The pairwise assessment demonstrated that the classifications of Sammartino et al. [11] and Chang et al. [8] exhibited a higher level of concordance (57.4%) (Figure 5) compared with both Sammartino et al. [11] and JD et al. [13] (46%) (Figure 6) and Chang et al. [8] and JD et al. [13] (39.3%) (Figure 7).

## 4. Discussion

This retrospective study aimed to assess the level of agreement between six distinct 3D classifications introduced by Juodzabalys and Daugela [13], Sammartino [11], Chang [8], Jhamb [10], Maglione [9], and Nemsi [12], concerning the difficulty score of the M3M. The study was conducted by performing two types of analysis, distinguishing between the classifications based on the assessment of a single parameter and those incorporating multiple items in the determination of a final difficulty score. The first analysis focused on the relationship between the M3M and the IAN, comparing the classifications of Maglione et al. [9], Nemsi et al. [12], A. Jhamb et al. [10], and the C parameter by Juodzabalys and Daugela et al. [13]. It revealed a low level of agreement among the classifications (26.1%), which was attributable to variations in the methodology used by the authors. Specifically, the classifications proposed by Jhamb et al. [10], Maglione et al. [9], and Nemsi et al. [12] evaluated the position of the mandibular canal (vestibular, lingual, or apical) and its distance from the wisdom tooth, while the JD et al. parameter C classification [13] was based only on the distance between the IAN and the M3M. The spatial orientation of the IAN relative to the wisdom tooth has been shown to be a crucial factor in determining the risk of IAN injury, and several authors have pointed out that a higher risk occurs when the mandibular canal is positioned lingually [19,20,21,22]. Although they followed a similar analytical principle, Jhamb et al. [10], Maglione et al. [9], and Nemsi et al. [12] used different scoring methods and class/subclass divisions in their classifications. To achieve the purpose of the study, score equalization was carried out by grouping the classes that presented similar scoring attribution criteria, without changing the criteria defined by each classification for the class definition. Indeed, in assessing the relationship between the M3M and the IAN, the various authors delineated different distances to establish the class divisions. This discrepancy became apparent in the pairwise comparison analysis, where the classifications by Nemsi et al. [12] and Jhamb et al. [10] exhibited the highest level of concordance (59.5%). The classifications were developed by evaluating the spatial relationship and distance (mm) between the IAN and the M3M, defining nearly identical thresholds between the classes. Both authors agreed that the risk of IAN injury increases dramatically as the distance between the two anatomical structures decreases, by defining similar cut-off distances (mm) between them, and that the greatest risk of sensory damage is associated with a partial/total loss of the cortical bone of the mandibular canal, with the exposure of the neurovascular bundle [10,12]. This was corroborated by Nakamori et al., who emphasized that the direct exposure of the neurovascular bundle and/or contact between the tooth and the IAN on CT scans increased the incidence of IAN injury by approximately 20% to 30% [23]. In addition to the distance between the M3M and the IAN, a correlation was established between the impaction patterns and inferior alveolar nerve injury, with the highest occurrence observed in horizontally impacted molars, followed by distally, mesially, and then vertically impacted molars [3,24,25,26]. In that regard, the depth of the third molar impaction has been directly correlated with the risk of inferior alveolar nerve injury [3,25].

In carefully planning the extraction of the lower third molar, it is reductive to focus on the relationships between the IAN and the M3M and it is imperative to consider the other parameters and structures contiguous to the M3M, as their damage could lead to significant complications [8,9,10,11,12,13,27]. The second analysis of this study compared the classifications of Juodzabalys and Daugela et al. [13], Sammartino et al. [11], and Chang et al. [8], who assessed the surgical difficulty of the lower third molar by simultaneously evaluating the angulation of the tooth and its mesio-distal, apico-coronal, and bucco-lingual positions. The angulation of the tooth may inevitably affect the surgical difficulty of the M3M extraction: a distolingual inclination determines a more difficult surgical access, and a heightened risk of complications, including migration within the floor of the mouth and potential damage to the lingual nerve [11]. The mesiodistal position also influences the surgical procedure, and it is determined in relation to the second molar and the mandibular ramus. The greatest risk of complications was observed when the tooth was fully impacted in the mandibular ramus, particularly when the M3Ms appear in a disto-angular or horizontal position [13]. The buccolingual position of the third molar in relation to the lingual and buccal walls of the mandible is indicative of the risk of lingual nerve injury. Iatrogenic lingual nerve injury may occur during the extraction of a third molar when it is in close proximity to the lingual wall, due to the proximity of the lingual nerve [11,13]. Analyzing the total difficulty score, the Sammartino et al. [11], Chang et al. [8], and JD et al. [13] classifications demonstrated a 25.5% agreement level, influenced by the methodology used to calculate the final total scores. Specifically, Sammartino et al. [11] and Chang et al. [8] calculated the final score as the sum of the scores assigned to each parameter, while JD et al. [13] evaluated the final score according to the highest value assigned among the various scores. As a result, when comparing the pairwise concordance levels, Sammartino et al. [11] and Chang et al. [8] demonstrated the highest concordance level of 57.4%, while Sammartino et al. [11] and JD et al. [13] showed a concordance rate of 46% and Chang et al. [8] and JD et al. [13] demonstrated 39.3%. The rationale behind the different criteria for calculating the final score was not defined by the authors. According to some authors, the more coherent approach involves calculating the final score using summation. Specifically, Stacchi et al. demonstrated that computing the final score as the sum of the scores assigned to each parameter, rather than selecting the highest value among the six parameters, resulted in a stronger correlation the of JD et al. [13] classification with the surgical time [28]. Another factor contributing to the low agreement among these classifications may be attributed to the variability in the parameters considered by different authors. Sammartino et al. [11] included bone density and wisdom tooth morphology in the classification. The presence of dense bone, especially in older patients, adds complexity to the surgical procedure due to reduced bone elasticity, making it difficult to differentiate the tooth from the surrounding bone. The bone density parameter is recognized as directly correlated with the patient’s age, which serves as a predictive factor in evaluating the surgical difficulty related to the lower third molar [11,29]. In addition, in order to obtain a comprehensive judgement parameter assessing the difficulty of M3M, it is necessary to evaluate the morphology of the lower third molar; no classification has included, but should include, root width, the number of roots, roots with abnormal curvature, and crown abnormalities in their evaluation. Diniz-Freitas et al. emphasized the importance of the root width and curvature as crucial factors, stating that scales for predicting the operative difficulty should include considerations of the root anatomy [30]. Akadiri and Obiechina also argued that, beyond the depth of the inclination of the wisdom tooth, root morphology is the most significant determinant of difficulty in lower third molar extraction [13,28]. For this reason, in order to obtain a comprehensive assessment method for lower third molar surgical difficulty, root characteristics should be contemplated.

Due to the high heterogeneity of the classification systems available in the literature, the main limitation of this study arises from the challenge of aligning the various systems considered. The process of equalizing the scores was therefore necessary to facilitate the comparison between the included methods, while maintaining the integrity of the underlying analysis criteria. It is also important to note that none of the proposed classifications have received formal validation through comprehensive clinical studies or established scientific consensus. The strength of the results of this study resides in the inclusion of a large study sample, which guarantees the reliability and validity of the findings. This not only minimizes the margin of error, but also increases the accuracy of the results. Furthermore, each image was viewed by four different investigators to ensure the robustness of the data.

## 5. Conclusions

This study highlights the current lack of a universally accepted and validated model to assess the difficulty of surgical procedures for lower third molars. The discrepancies that have emerged among the existing classifications represent a potential risk of error in determining optimal surgical planning for M3M cases. Further investigation is imperative to develop a comprehensive and unbiased multiparametric classification approach. The future aspiration is to provide an objective, automated, and operator-independent method for assessing the surgical difficulty associated with lower third molars.

## Figures and Tables

**Table 1:** Classification proposed by Maglione [9].

CLASS Relationship between the IAN and the M3M in the Buccolingual Section	SUBCLASSES Distance between the IAN and the M3M	
Class 0: the mandibular canal is not visible on the image (plexiform canal)		
Class 1: the mandibular canal runs apically or buccally without touching the tooth	1A	distance greater than 2 mm
	1B	distance less than 2 mm
Class 2: the mandibular canal runs lingually without touching the tooth	2A	distance greater than 2 mm
	2B	distance less than 2 mm
Class 3: the mandibular canal runs apical or buccal touching the tooth	3A	without interruption of the corticalization
	3B	with interruption of the corticalization
Class 4: the mandibular canal runs lingually touching the tooth	4A	without interruption of the corticalization
	4B	with interruption of the corticalization
Class 5: the mandibular canal runs between the roots but without touching them	5A	distance greater than 2 mm
	5B	distance less than 2 mm
Class 6: the mandibular canal runs between the roots touching them	6A	without interruption of the corticalization
	6B	without interruption of the corticalization
Class 7: the mandibular canal runs between the fused roots		

**Table 2:** Classification proposed by Nemsi [12].

CLASS	SUBCLASSES (Score)
Class A: the mandibular canal is apical to the M3M root	The mandibular canal is distant more than 1.5 mm from the roots (1). The mandibular canal is distant less than 1.5 mm from the roots with the total presence of its cortical lining (2). The mandibular canal is distant less than 1.5 mm from the roots with total or partial loss of its cortical lining with preserved calibre (3). Direct contact with a reduced calibre of the mandibular canal (4).
Class B: the mandibular canal is buccal to the M3M root	
Class L: the mandibular canal is lingual to the M3M root.	

**Table 3:** Classification proposed by Jhamb [10].

Distance between M3M and IAN	Score
>1 mm	1
0–1 mm	2
0 mm with cortication	3
0 mm with a cortical break	4

**Table 4:** Classification proposed by JD [13].

Position of the Mandibular Third Molar	Risk Degree of Presumptive Intervention (Score)			
	Conventional (0)	Simple (1)	Moderate (2)	Complicate (3)
Mesiodistal position in relation to the second molar—M and the mandibular ramus—R				
Relation to the second molar—M	Crown directed at or above the equator of the second molar	Crown directed below the equator to the coronal third of the second molar root	Crown/roots directed to the middle third of the second molar root	Crown/roots directed to the apical third of the second molar root
Relation to the mandibular ramus—R	Sufficient space in the dental arch	Partially impacted in the ramus	Completely impacted in the ramus	Completely impacted in the ramus in the distolingual or horizontal position
Apicoronal position in relation to the alveolar crest—A and the mandibular canal—C				
Relation to the adjacent alveolar crest (from the uppermost point of the tooth)—A	Tooth is completely erupted	Partially impacted, but widest part of the crown (equator) is above the bone	Partially impacted, but the widest part of the crown (equator) is below the bone	Completely encased in the bone
Relation to the mandibular canal (from the lowermost point of the tooth)—C	≥3 mm to the mandibular canal	Contacting or penetrating the mandibular canal, wall of the mandibular may be identified	Contacting or penetrating the mandibular canal, wall of the mandibular may be identified	Roots surrounding the mandibular canal
Buccolingual position in relation to the mandibular lingual and buccal walls—B (LN injury risk)				
Relation to the mandibular lingual and buccal walls—B	Closer to the buccal wall	In the middle between the lingual and buccal walls	Closer to the lingual wall	Closer to the lingual wall, when the tooth is partially impacted or completely encased in the bone (A2 o A3)
Spatial position—S				
Spatial position	Vertical (90°)	Mesioangular (≤60°)	Distoangular (≥120°)	Horizontal (0°) or inverted (270°)
FINAL SCORE: For each parameter, the score ranged from 0 to 3, and the presumed degree of the risk of surgery will be defined by the highest value among the parameters considered.				

**Table 5:** Classification proposed by Sammartino [11].

	DEFINITION	SCORE
ANGULATION	Inclination of the third molar	1–5
AVAILABLE SPACE	Three different classes	1–3
DEPTH	Three different apico-coronal positions	1–3
RELATION WITH THE MANDIBULAR CANAL	Three different third molar positions in relation to the mandibular canal	1–3
BONE DENSITY	Four different bone densities	1–4
BUCCOLINGUAL POSITION	Three different positions in relation to the buccal or lingual cortical bone	1–3
DENTAL MORPHOLOGY	Two different classes in relation to the third molar morphology	1–2
FINAL SCORE: THE SUM OF THE SCORES OF THE ANALYZED PARAMETERS ALLOWED US TO CLASSIFY THE DIFFICULTY OF THE LOWER THIRD MOLAR EXTRACTION INTO THREE CLASSES		LOW: 6.5–12.5 points
		MEDIUM: 13–17.5 points
		HIGH: 8–22 points

**Table 6:** Classification proposed by Chang [8].

	DEFINITION	SCORE
SPATIAL RELATIONSHIP	Inclination of the third molar	1–5
DEPTH	Four different apico-coronal positions	1–4
RAMUS RELATIONSHIP/SPACE	Considering both the distance between the distal surface of the second molar and the mandibular ramus, and the diameter of the third molar	1–3
FINAL SCORE: THE DIFFICULTY INDEX WAS CALCULATED AS THE SUM OF THE SCORES CONSIDERED		I: 3–4 points
		II: 5–7 points
		III: 8–10 points
		IV: 11–12 points

**Table 7:** Score equalization method of the classifications proposed by Maglione et al. [9], Nemsi et al. [12], Jhamb et al. [10], and the C-parameter of Juodzabalys and Daugela et al. [13]. In the classification of Maglione et al. [9]: (1) the first and second classes were merged because in both cases the mandibular canal runs without touching the root; (2) the third and fourth classes, including the tooth in contact with the NAI, have been distinguished according to the location of the nerve in the lingual or buccal direction. The increased risk of neurological complications arises when the nerve is positioned lingually in relation to the wisdom tooth [19]; (3) the fifth, sixth, and seventh classes emphasized the course of the mandibular canal between the roots of the lower third molar; for this reason, they have been grouped into a single class. In the Nemsi et al. [12] classification, we combined the fourth subclass and uncommon classes (U) because they all identified a close IAN/M3M relationship.

SCORE				FINAL SCORE
Maglione	Nemsi	JD	Jhamb	
1–2	1	0	1	1
3	2	1	2	2
4	3	2	3	3
5–6–7	4 + U	3	4	4

**Table 8:** Score equalization method of the classifications proposed by Juodzabalys and Daugela et al. [13], Sammartino et al. [11], and Chang et al. [8]. In the Chang et al. [8] M3M classification, the categories with a total degree of difficulty of III and IV were combined into a single class (High-3) because they indicated complex and uncommon anatomical conditions. Regarding the JD et al. [13] classification, levels 1 and 2 were combined because of the presence of parameters with highly related characteristics, with an almost similar surgical risk.

SCORE			FINAL SCORE
Sammartino	Chang	JD	
6.5–12.5	3–4	0	Low (1)
13–17.5	5–7	1–2	Medium (2)
18–22	8–12	3	High (3)

**Table 9:** Descriptive statistics analysis: absolute frequencies of 3D classifications regarding the M3M position with the IAN and for the evaluation of the overall difficulty score considering multiple parameters.

3D Classification that Evaluated the Relationship between M3M Roots and IAN																			
Score Difficulty				Maglione				Nemsi				Jhamb				JD			
1				212				143				159				160			
2				158				151				42				165			
3				115				102				137				133			
4				36				125				183				63			
**3D classification for the assessment of the overall difficulty score considering the angulation of the tooth, and its mesio-distal, apico-coronal and bucco-lingual positions**																			
Score Difficulty					JD					Sammartino					Chang				
1					79					212					171				
2					300					286					329				
3					142					14					21				

## Data Availability

Data will be available from the corresponding authors.
